# Respiratory effort during sleep predicts mortality in patients with suspected obstructive sleep apnea

**DOI:** 10.1093/ajrccm/aamag344

**Published:** 2026-06-29

**Authors:** Hamza Nahoui, Henrik Schirmer, Gunnar Einvik, Tarjei Øvrebotten, Kristian Berge, Peder L Myhre, Haldor Husby, Harald Hrubos-Strøm

**Affiliations:** Department of Cardiology, Division of Medicine, Akershus University Hospital, Lørenskog, Norway; Institute of Clinical Medicine, University of Oslo, Oslo, Norway; Department of Cardiology, Division of Medicine, Akershus University Hospital, Lørenskog, Norway; Institute of Clinical Medicine, University of Oslo, Oslo, Norway; Institute of Clinical Medicine, University of Oslo, Oslo, Norway; Department of Pulmonary Diseases, Division of Medicine, Akershus University Hospital, Lørenskog, Norway; Department of Cardiology, Division of Medicine, Akershus University Hospital, Lørenskog, Norway; Institute of Clinical Medicine, University of Oslo, Oslo, Norway; Department of Cardiology, Division of Medicine, Akershus University Hospital, Lørenskog, Norway; Institute of Clinical Medicine, University of Oslo, Oslo, Norway; Department of Cardiology, Division of Medicine, Akershus University Hospital, Lørenskog, Norway; Institute of Clinical Medicine, University of Oslo, Oslo, Norway; Department of Cardiology, Oslo University Hospital Rikshospitalet, Oslo, Norway; Deparment of Data and Analytics, Akershus University Hospital, Lørenskog, Norway; Institute of Clinical Medicine, University of Oslo, Oslo, Norway; Department of Ear, Nose and Throat, Division of Surgery, Akershus University Hospital, Lørenskog, Norway

**Keywords:** respiratory effort, obstructive sleep apnea, esophageal pressure, all-cause mortality, sleep-disordered breathing

## Abstract

**Rationale:**

Obstructive sleep apnea (OSA) confers increased risk of morbidity and mortality through mechanisms like exaggerated negative intrathoracic pressure and hypoxemia.

**Objectives:**

Evaluate the association between negative esophageal pressure (PES; a surrogate of respiratory effort) during sleep and long-term all-cause mortality in suspected OSA patients.

**Methods:**

Patients referred with suspected OSA (2003-2016) underwent overnight respiratory polygraphy with PES monitoring. PES, mean oxygen saturation (mSpO_2_), apnea-hypopnea index (AHI), total time in bed (TiB), and time in bed spent above different PES thresholds (10-60 cmH_2_O) were recorded. Clinical data and mortality until June 2024 were obtained from medical records. Mortality was analyzed using multivariable Cox regression.

**Results:**

In 16 083 patients (26% female; median age 48 years), median PES was 12.8 cmH_2_O (IQR 8.7-18.4). Median mSpO_2_ was 94% (93%-95%), and median AHI was 10 (4.3-21.1); 16% had AHI ≥ 30. During median follow-up of 15 years (IQR 12-18), 1497 (9.3%) died. Patients in the third and fourth quartiles of median PES had increased mortality risk compared to the first quartile. After adjustment for age, sex, body mass index (BMI), comorbidities, AHI, and mSpO_2_, median PES remained associated with mortality, with the third quartile showing increased risk (hazard ratio [HR] 1.21; 95% CI, 1.03-1.43; *P* = .024). Longer time spent above higher PES thresholds was associated with increased mortality. Risk rose across quartiles of percentage of TiB at each threshold and increased further with more negative thresholds in the highest quartile.

**Conclusion:**

Respiratory effort, measured by PES during sleep, independently predicts all-cause mortality, and provides prognostic information beyond traditional OSA metrics including AHI and hypoxemia. This supports respiratory effort as a useful component of comprehensive clinical evaluation.

At a Glance Commentary **Scientific Knowledge on the Subject:** Exaggerated negative intrathoracic pressure during obstructive events is a core mechanism of harm in obstructive sleep apnea (OSA). Even though esophageal pressure (PES) is the gold standard for estimating intrathoracic pressure and respiratory effort, it is invasive and rarely measured. Prior mortality studies therefore have characterized OSA almost entirely through the apnea-hypopnea index (AHI) and hypoxemia, and the long-term mortality impact of directly measured respiratory effort during sleep has been unknown.

**What This Study Adds to the Field:** In 16 083 patients with suspected OSA followed for a median of 15 years, greater respiratory effort (measured by median PES through the night) independently predicted all-cause mortality after adjustment for age, sex, BMI, comorbidities, AHI, and mean nocturnal oxygen saturation. Both the depth and the duration of effort contributed in a dose-dependent manner, and the prognostic value was most evident in nonsevere OSA. Respiratory effort conveys prognostic information beyond AHI and hypoxemia, supporting a more comprehensive assessment of OSA severity.

## Introduction

Sleep is 1 of 8 essential factors for cardiac health according to the American Heart Association.^1^ Obstructive sleep apnea (OSA) is a common sleep disorder^2^ affecting almost 1 billion people worldwide.^3^ Severe OSA, defined by an apnea-hypopnea index (AHI) ≥ 30, is linked to high mortality rates and cardiovascular comorbidities.^3-5^ Yet treating OSA by positive airway pressure (PAP) has not had the anticipated effects on mortality or cardiovascular comorbidity.^6^ Therefore further exploration of physiological markers and disease mechanisms to capture the pathophysiological burden of OSA and mortality risk is warranted.

Exaggerated negative intrathoracic pressure (respiratory effort) and hypoxemia during obstructive episodes are 2 important pathophysiological mechanisms of OSA and might provide prognostic information beyond AHI alone.^4^ Negative intrathoracic pressure affects cardiac function negatively by several mechanisms. It increases left ventricle transmural pressure and venous blood return, causing increased left ventricular afterload and preload respectively.^7^ By using an esophageal catheter, clinicians can estimate the intrathoracic pressure (respiratory effort) by measuring the negative esophageal pressure (PES), which correlates closely to both pleural pressure and pericardial pressure.^8^ Nocturnal hypoxemia, induced by prolonged obstructive episodes, also may contribute to physiological stress through pulmonary vasoconstriction, oxidative stress, inflammation, and endothelial dysfunction.^4^^,^^9^^,^^10^ Post-apneic reoxygenation generates reactive oxygen species that diminish nitric oxide levels and impair endothelially mediated vasodilation predisposing to hypertension.^10^

To our knowledge, no previous studies have examined the association between negative esophageal pressure during sleep and long-term mortality. In this study, we hypothesize that greater negative esophageal pressure is independently associated with increased risk of mortality. Additionally, we aimed to investigate the associations of traditional sleep metrics AHI and mean nocturnal SpO_2_ with mortality in the same cohort.^11^^,^^12^

## Methods

### Design and patients

This was a retrospective observational study on a sample of 16 083 patients referred to Akershus University Hospital (AUH) with suspected OSA between 2003 and 2016. All patients performed in-hospital overnight respiratory polygraphy with simultaneous esophageal and oropharyngeal pressure measurements as part of the physiological assessment to determine whether upper airway collapse contributed to obstruction and to identify potential candidates for upper airway surgery. Patients older than 90 years and younger than 18 years were excluded.

### Data collection

The date of in-hospital respiratory polygraphy was defined as the baseline of this study. Data from the polygraphy (Apneagraph 200, Bergen, Norway) included: (1) airflow from a nasopharyngeal catheter with 2 thermistors; (2) PES from an esophageal catheter; and (3) SpO_2_ and pulse from a peripheral finger plethysmograph.

A Millar-type catheter (non-balloon) was used to monitor esophageal pressure during sleep. The catheter was 90 cm long, with an outer diameter of 2.0 ± 0.2 mm and a soft distal tip to facilitate transnasal insertion. It contained 2 solid-state pressure sensors: a proximal sensor located 30 cm from the catheter tip, intended to be positioned in the oropharynx at the level of the velum, and a distal sensor located 5 cm from the tip, intended for pressure measurement in the esophagus. According to the catheter specifications, a marking ring was located 26.7 cm from the catheter tip and was used as part of the local placement protocol. The catheter was inserted transnasally by trained nurses in the sleep clinic at the Department of Otorhinolaryngology at Akershus University Hospital. Placement was guided by the catheter marker, and the catheter was advanced until this marker was positioned approximately 3 cm from the nasal tip, after which it was secured for overnight monitoring. No formal physiological validation of catheter position or signal quality was performed before securing the catheter, and insertion depth was not adjusted for patient height. Additional technical details and figures regarding the catheter design and sensor configuration are provided in the supplementary material and Figures S1, S2, S3 (see online supplementary material for these figures). Automatic removal of swallow artifacts was implemented in the software used in the polygraphy analysis.

Polygraphy data were primarily analyzed by the Apnea analysis or Spiro analysis software (both Bergen, Norway). The analysis time was defined between self-reported lights off and on, after removal of artifacts. The following variables were extracted for the purpose of this study: total time in bed (TiB), TiB with elevated peak-to-peak PES 5 to 95 cmH_2_O in increments of 5 cmH_2_O, mean SpO_2_, and AHI. Body mass index (BMI) was registered by nurses in the sleep section on the day of the respiratory polygraphy.

Median PES was defined as the pressure level above which a patient spent 50% of their total TiB. The data set contained predefined intrathoracic pressure thresholds ( > 10, >15, . . ., >95 cmH_2_O) and the corresponding percentage of TiB spent above each threshold. Because this percentage decreases monotonically with increasing pressure, the exact 50% point typically fell between 2 thresholds. We used linear interpolation to estimate the continuous PES value corresponding to the 50% time point using the formula:^13^  $\;\mathrm{Median}\;\mathrm{PES}=P1+(\frac{T1-50}{T1-T2})X(P2-P1)\;$ where *P*1 was the lower threshold when %TiB ≥ 50%, *T*1 was the %TiB above *P*1, *P*2 was the next higher threshold with %TiB < 50%, and *T*2 was the corresponding percentage. This method yielded a patient-specific continuous estimate of median PES.

We used mean nocturnal oxygen saturation (mSpO_2_) to measure nocturnal hypoxemia, as its clinical relevance had been investigated previously.^14^^,^^15^ Hypoxemia was defined as mSpO_2_ less than 92%, in agreement with previous research.^16^ AHI was measured as the number of obstructive apneas and hypopneas (10 seconds or longer) per hour for the whole night combined with more than 4% desaturation related to the apnea/hypopnea episode. AHI < 5 was defined as non-OSA, AHI 5-15 was defined as mild OSA, AHI 15-30 was defined as moderate OSA, and AHI ≥ 30 was defined as severe OSA.

Patient demographics were obtained from medical records. Predefined comorbidities were identified at baseline using the International Classification of Diseases, 10th revision (ICD-10). Codes were recorded before or at the time of the diagnostic sleep study. ICD-9 codes were not used. Diagnostic codes were obtained from the electronic medical records at Akershus University Hospital and included codes registered from all hospital contacts, including discharge reports and outpatient visits. The comorbidities assessed were heart failure, atrial fibrillation, myocardial infarction, ischemic heart disease, stroke, chronic obstructive pulmonary disease (COPD), chronic kidney disease (CKD), hypertension, diabetes, anemia, cancer, and dementia (Table S1; see online supplementary material for table).

Mortality data were retrieved from electronic medical records at Akershus University Hospital, which was updated from the Norwegian Cause of Death Registry by June 2024. The study was approved by the Regional Committee for Health Research Ethics and the requirement for informed consent was waived. Information on treatment after diagnostic examination (respiratory polygraphies), including PAP use and adherence, was not available for this study.

### Statistical analysis

Normality of continuous variables was assessed with histograms. Continuous variables are presented as median with interquartile range (IQR: Q1-Q3), depending on distribution. Categorical variables are reported as absolute numbers and percentages. Baseline characteristics across quartiles of median PES were compared using Cuzick’s nonparametric test for trend for skewed continuous variables and the chi-square test for categorical variables.

To investigate the association between sleep metrics and all-cause mortality, we performed survival analysis using Cox proportional hazards models, with time from sleep examination to death or censoring (maximum 20 years) as the time scale. Median PES was categorized into quartiles (Q1, 2, 3, 4). AHI was categorized based on clinical cutoffs: no OSA (AHI < 5), mild (5-14.9), moderate (15-29.9), and severe OSA (≥30). Mean SpO_2_ was categorized as < 92%, 92-95%, and > 95%, according to prior recommendations.^16^

Three Cox models were then constructed for quartiles of median PES. Model 1 adjusted for age, sex, and BMI. Model 2 additionally included comorbidities (heart failure, atrial fibrillation, myocardial infarction, ischemic heart disease, stroke, COPD, CKD, hypertension, diabetes, anemia, cancer, and dementia). Model 3 further adjusted for the other 2 sleep metrics (AHI and mean SpO_2_). Subgroup analyses included stratification by AHI (<vs ≥ 30), age (<vs ≥ 50 years) and sex. A sensitivity analyses included follow-up, truncated at maximum 10 years. Secondary analyses evaluated AHI categories and mean SpO_2_ categories as primary exposures using the same 3-stage modeling strategy.

To further characterize the relationship between intrathoracic pressure swings and mortality, we conducted a series of Cox proportional hazards models using quartiles of the percentage of time in bed spent above increasing PES thresholds, ranging from > 10 to > 60 cmH_2_O. Each model was adjusted for age, sex, BMI, and comorbidities (Model 2).

Assumptions of proportional hazards were tested using Schoenfeld residual plots.^17^ Collinearity was assessed using variance inflation factor (VIF). Linearity of continuous variables was evaluated using Martingale residual plots.^18^ Concordance index (Harrell’s C-index) was used to assess the discriminatory ability of the Cox models, and likelihood ratio tests and Akaike’s information criterion (AIC) were used to compare model fit and evaluate the predictive value of sleep variables.

To flexibly model nonlinear associations, restricted cubic splines with 3 knots were used for each sleep metric in adjusted Cox models (including spline terms for age, BMI, AHI, mean SpO_2_, and comorbidities).^19^ Spline models were used to visualize the continuous effect of median PES and AHI on mortality. Statistical analyses were conducted with Stata18.0.

## Results

### Descriptive statistics

Of 16 083 analyzed patients, 4227 (26%) were female, and the median age was 48 (IQR 40-57) years. The median AHI was 10 (IQR 4.5-21.3) and 11 673 (72.5%) were classified with OSA, including 5887 (37%) with mild OSA, 3167 (19.6%) with moderate OSA, and 2592 (16%) with severe OSA. The median mSpO_2_ was 94% (93%–95%) (Table 1). The median PES was 12.8 cmH_2_O (IQR 8.7-18.4) with a right-skewed distribution (Figure S4, see online supplementary material for this figure). The fourth quartile included values up to 77.7 cmH_2_O, indicating a subgroup with markedly elevated negative esophageal pressure during bedtime. Patients in the third and fourth quartiles were older (Q3: 50 [IQR 42-59] years; Q4: 50 [42-58]) compared to those in the first and second quartiles (Q1: 45 [36-55]; Q2: 48 [40-57]) (Table 1). BMI increased progressively across quartiles of median PES from a median of 25.8 [IQR 23.9-28.1] in Q1 to 32.2 [29.1-36.1] in Q4. Proportion of women increased across PES-quartiles, indicating that higher respiratory effort was more frequently observed among women in this cohort (24.1% and 25.3% in Q1 and Q2 vs 26.9% and 28.8% in Q3 and Q4) (Table 1).

**Table 1:** Baseline characteristics of the study cohort across quartiles of median intrathoracic pressure (PES) during sleep.

	Q1 median PES (2.5-8.7 cmH_2_O)	Q2 median PES (8.7-12.8 cmH_2_O)	Q3 median PES (12.8-18.4 cmH_2_O)	Q4 median PES (18.4-77.7 cmH_2_O)	Total (*n* = 16 083)	*P* value
**Age, median (IQR)**	45 (36-55)	48 (40-57)	50 (42-59)	50 (42-58)	48 (40-57)	<.001
**BMI, median (IQR)**	25.8 (23.9-28.1)	27.7 (25.5-30.1)	29.4 (27-32.4)	32.2 (29.1-36.1)	28.6 (25.7-32)	<.001
**AHI, median (IQR)**	4.8 (2.4-9.5)	7.5 (3.8-14.2)	11.5 (6-21)	25.2 (12.5-45.5)	10 (4.5-21.3)	<.001
**Mean SpO_2_, median (IQR)**	95.1 (94.2-95.9)	94.6 (93.6-95.4)	94.1 (93-95)	93.3 (91.9-94.4)	94.3 (93.1-95.3)	<.001
**Female, *n* (%)**	970 (24.1)	1017 (25.3)	1082 (26.9)	1158 (28.8)	4227 (26.3)	<.001
**Heart failure, *n* (%)**	74 (1.8)	101 (2.5)	143 (3.6)	187 (4.7)	505 (3.1)	<.001
**Atrial fibrillation, *n* (%)**	46 (1.1)	52 (1.3)	59 (1.5)	55 (1.4)	212 (1.3)	.632
**Myocardial infarction, *n* (%)**	33 (0.8)	44 (1.1)	61 (1.5)	80 (2.0)	218 (1.4)	<.001
**Ischemic heart disease, *n* (%)**	90 (2.2)	118 (2.9)	166 (4.1)	181 (4.5)	555 (3.5)	<.001
**Stroke, *n* (%)**	49 (1.2)	50 (1.2)	66 (1.6)	55 (1.4)	220 (1.4)	.340
**COPD, *n* (%)**	26 (0.6)	35 (0.9)	36 (0.9)	50 (1.2)	147 (0.9)	.044
**Chronic kidney disease, *n* (%)**	16 (0.4)	12 (0.3)	33 (0.8)	31 (0.8)	92 (0.6)	.002
**Hypertension, *n* (%)**	136 (3.4)	175 (4.4)	272 (6.8)	324 (8.1)	907 (5.6)	<.001
**Diabetes, *n* (%)**	56 (1.4)	66 (1.6)	128 (3.2)	160 (4.0)	410 (2.5)	<.001
**Anemia, *n* (%)**	21 (0.5)	23 (0.6)	18 (0.4)	28 (0.7)	90 (0.6)	.499
**Cancer, *n* (%)**	50 (1.2)	52 (1.3)	60 (1.5)	63 (1.6)	225 (1.4)	.550
**Dementia, *n* (%)**	3 (0.1)	1 (0.0)	1 (0.0)	1 (0.0)	6 (0.0)	.572

*P* values are descriptive and reflect unadjusted comparisons across PES quartiles. They were not used for statistical inference in the analysis.

Abbreviations: AHI, apnea-hypopnea index; BMI, body mass index; COPD, chronic obstructive pulmonary disease; IQR, interquartile range; PES, negative esophageal pressure; Q, quartile.

Conversely, mean SpO_2_ decreased with increasing median PES: 95% [94-96] in Q1 and 93% [92-94] in Q4. AHI was higher in patients with higher PES quartiles: 4.8 [2.4-9.5] in Q1 to 25.2 [12.5-45.5] in Q4, suggesting that greater respiratory effort during sleep was associated with both more severe hypoxemia and sleep apnea (Table 1). These associations were further supported by moderate correlations between median PES and both AHI (r = 0.60) and mean SpO_2_ (r = –0.44). The relationship between median PES and each of AHI and mean SpO_2_ is illustrated in the supplementary Figures S5 and S6 (see online supplementary material for these figures).

### Association with all-cause mortality

During a median follow-up of 15 years [IQR: 12-18], 1497 patients (9.3%) died. Compared to survivors, nonsurvivors were older (61 [53-69] vs 47 [39-56] years), more likely to be male (76.8% vs 73.4%), and had higher median PES (14.3 [9.9-19.5] vs 12.7 [8.6-18.2] cmH_2_O), higher AHI (16.8 [7.7-31.5] vs 9.5 [4.3-20.3]), and lower mean SpO_2_ (93.3% [91.9-94.5] vs 94.4% [93.2-95.4]) (Table S2; see online supplementary material for this table).

Compared to patients in the first quartile of median PES (<8.7 cmH_2_O), those in the third and fourth quartiles had significantly higher risk of all-cause mortality. In the age-, sex-, and BMI-adjusted model (Model 1), HRs were 1.32 (95% CI, 1.12-1.55; *P* = .001) and 1.37 (95% CI, 1.15-1.62; *P* < .001), respectively. This association remained significant after additional adjustment for comorbidities (Model 2), with HRs of 1.29 (95% CI, 1.11-1.53; *P* = .001) and 1.37 (95% CI, 1.15-1.62; *P* < .001). In the fully adjusted model (Model 3), which additionally included AHI and mean SpO_2_ categories, the association persisted for the third quartile (HR 1.21; 95% CI, 1.03-1.43; *P* = .024). Although there was a clear trend toward an association between the fourth quartile of median PES and mortality in the fully adjusted Model 3, the association did not reach statistical significance (HR 1.17; 95% CI, 0.97-1.41; *P* = .102) (Table 2; Figure S7, see online supplementary material for this figure). Model 3 demonstrated improved model performance compared to Model 2, as demonstrated by lower AIC and a likelihood ratio (LR) test (*P* < .001). Spline modeling demonstrated a significant overall association between median PES and mortality in the fully adjusted model (*P* = .023) (Figure 1). The relationship was nonlinear as indicated by a significant nonlinear spline component (*P* = .016) (Figure 1).

**Figure 1:**
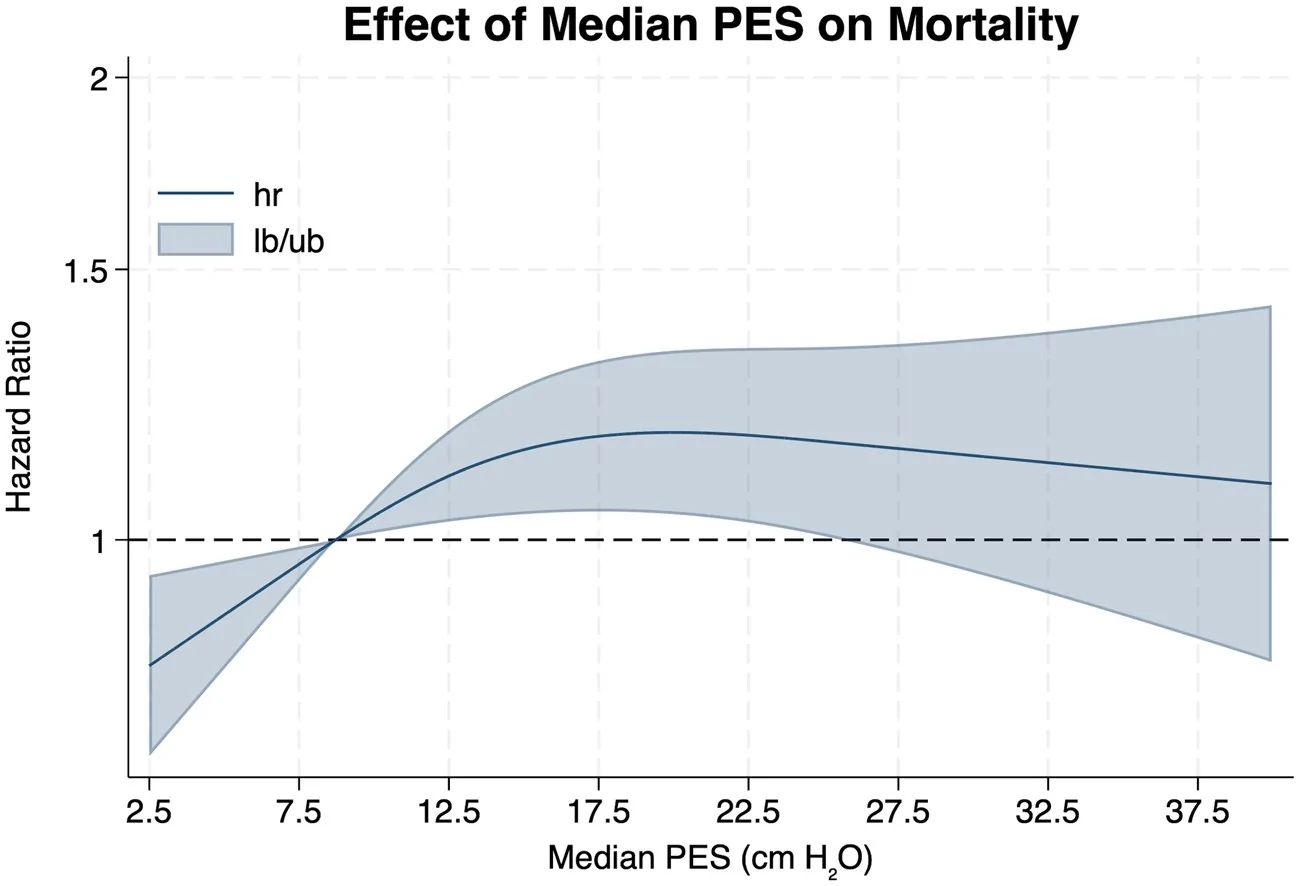
Association between median negative esophageal pressure (PES) and all-cause mortality using restricted cubic spline modeling. The figure displays the adjusted hazard ratio (HR; solid line) and 95% confidence intervals (CIs; shaded area) for mortality across the distribution of median PES (cmH_2_O), modeled using restricted cubic splines. The reference value was set at the 25th percentile of PES (8.7 cmH_2_O), indicated by the intersection with the horizontal HR = 1 line. The association between PES and mortality appears approximately linear up to ∼18 cmH_2_O, after which the curve plateaus, suggesting diminishing incremental risk at very high PES levels. The model was adjusted for age, sex, BMI, AHI, mean SpO_2_, and comorbidities. All continuous variables, including median PES, AHI, mean SpO_2_, age, and BMI, were modeled as spline variables to account for nonlinear effects. Abbreviations: AHI, apnea-hypopnea index; BMI, body mass index; SpO_2_, oxygen saturation.

**Table 2:** Association between quartiles of median PES and all-cause mortality across 3 adjustment models.

Median PES quartile	Model 1 (Adjusted for age, sex, BMI)	Model 2 (Model 1 + comorbidities)	Model 3 (Model 2 + AHI & SpO_2_)
**Q2 vs Q1**	1.12 (0.95-1.32), *P* = .179	1.15 (0.97-1.35), *P* = .100	1.13 (0.96-1.34), *P* = .141
**Q3 vs Q1**	1.32 (1.12-1.55), *P* = .001	1.29 (1.11-1.53), *P* = .001	1.21 (1.03-1.43), *P* = .024
**Q4 vs Q1**	1.37 (1.15-1.62), *P* < .001	1.37 (1.15-1.62), *P* < .001	1.17 (0.97-1.41), *P* = .102

Abbreviations: AHI, apnea-hypopnea index; BMI, body mass index; Q, quartile; SpO_2_, oxygen saturation.

In subgroup analyses stratified by OSA severity, the association between median PES quartiles and mortality was attenuated in patients with AHI ≥ 30, suggesting reduced prognostic value of PES in the context of severe OSA (HR 0.74; 95% CI, 0.49 − 1.10; *P* = .136) for patients in Q3 (Table S3; see online supplementary material for this table). In contrast, median PES was strongly associated with mortality risk in both the third (HR 1.32; 95% CI, 1.10-1.59; *P* = .003) and fourth quartiles (HR 1.35; 95% CI, 1.09-1.68; *P* = .006) in patients with AHI < 30 (Table S3). Subgroup analysis stratified by age, sex, and sensitivity analysis censoring at 10 years of follow-up are presented in Table S4 and Table S5 (see online supplementary material for these tables).

In a secondary analysis examining AHI as the main exposure, severe OSA (AHI ≥ 30) was significantly associated with mortality in Model 1 (HR 1.26; 95% CI, 1.05-1.51; *P* = .010) and Model 2 (HR 1.23; 95% CI, 1.03-1.47; *P* = .024), but not in Model 3 (HR 1.03; 95% CI, 0.85-1.26; *P* = .721) when adding mSpO_2_ and median PES (Table S6, Figure S8; see online supplementary material for this table and this figure). Mean SpO_2_ < 92% was associated with higher mortality across all models. Compared to patients with SpO_2_ > 95%, HRs were 2.06 (95% CI, 1.72-2.47; *P* < .001) in Model 1, 1.95 (95% CI, 1.62-2.35; *P* < .001) in Model 2, and 1.82 (95% CI, 1.50-2.20; *P* < .001) in Model 3 (Table S7; see online supplementary material for this table).

In exploratory analyses investigating associations between percentage of TiB spent with PES > 10 to > 60 cmH_2_O, we observed that higher quartiles of %TiB exposure were associated with increased mortality risk at each PES threshold (Figure 2;  Table S8, see online supplementary material for this table). This association was most pronounced in the fourth quartile, where hazard ratios increased with increasing pressure thresholds. For instance, at the > 10 cmH_2_O threshold, the hazard ratio (HR) for fourth quartile vs first quartile was 1.38 (95% CI, 1.17-1.64), whereas at > 30 cmH_2_O, the corresponding HR increased to 1.54 (95% CI, 1.30-1.83), and further to 1.66 (95% CI, 1.43-1.92) at > 50 cmH_2_O (Table S8), suggesting that both the intensity (increasing PES thresholds) and duration (greater %TiB in higher quartiles) of negative esophageal pressure were incrementally associated with higher mortality risk.

**Figure 2:**
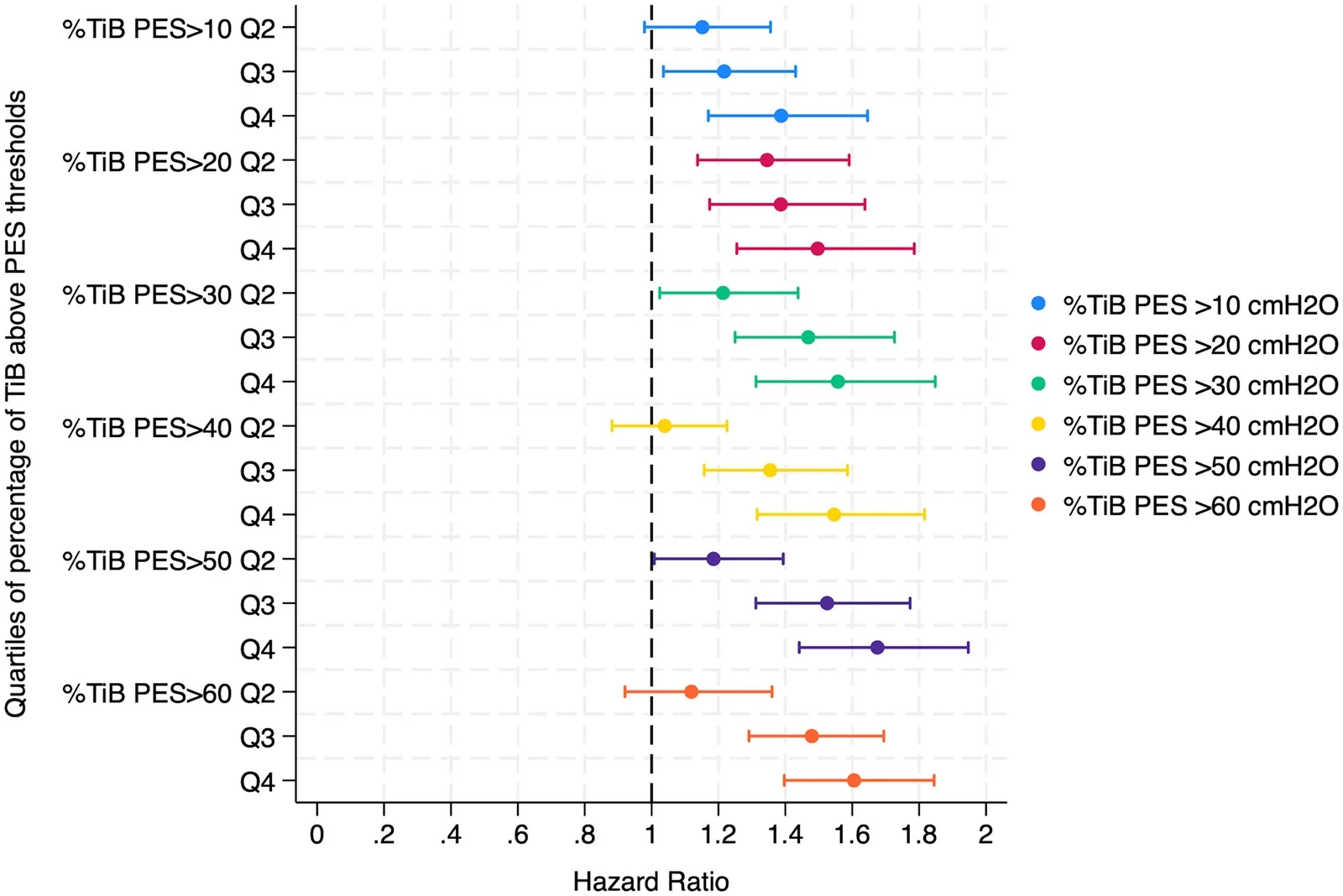
Association between quartiles of percentage of time in bed (%TiB) with negative intrathoracic pressure (PES) above increasing thresholds and all-cause mortality. Hazard ratios (HRs) and 95% confidence intervals (CIs) are shown for quartiles (Q2-Q4) of the percentage of TiB spent above PES thresholds ranging from > 10 to > 60 cmH_2_O, using Q1 (lowest quartile) as the reference. Estimates are based on a series of Cox regression models adjusted for age, sex, BMI, and comorbidities (Model 2). Within each threshold, mortality risk increased progressively across quartiles. Moreover, HRs were consistently higher at more extreme PES thresholds, particularly among patients in the highest quartile (Q4). Abbreviation: BMI, body mass index.

## Discussion

In this large retrospective real-world cohort of patients with suspected OSA with long-term follow-up, we have demonstrated that respiratory effort during sleep, quantified by median PES, is independently associated with all-cause mortality. This association remained significant after adjustment for age, sex, BMI, comorbidities, mean nocturnal oxygen saturation, and AHI. Notably, the prognostic significance of negative intrathoracic pressure extends beyond the median PES value. A dose-dependent increase in mortality risk was observed across quartiles of percentage of time in bed spent above increasing PES thresholds, suggesting that both the depth and duration of respiratory effort are relevant to long-term outcomes.

PES is the gold standard to estimate the intrathoracic pressure and the respiratory effort.^20^ Its close relation to pleural pressure and pericardial pressure^8^^,^^21^ allows calculating transpulmonary pressure and left ventricle transmural pressure (transpulmonary pressure = alveolar pressure minus pleural pressure).^21^^,^^22^ Eso­phageal pressure has long been used in mechanistic studies as a surrogate for intrathoracic pressure. Important work by Buda  et al,^23^ and later by Naughton  et al,^24^ demonstrated that exaggerated negative intrathoracic pressure, reflected by increased PES, can impair cardiac function,^23^ and that patients with chronic heart failure exhibit elevated PES even during wakefulness.^24^ Building on these experimental findings, our study extends the relevance of PES into a real-world clinical context, showing that increased respiratory effort, as measured by median PES, is an independent and clinically meaningful predictor of all-cause mortality in patients with suspected OSA.

Subgroup analyses of this study provided additional insight into the contexts in which negative esophageal pressure carries prognostic value. In patients with severe OSA (AHI ≥ 30), the association between median PES and mortality was significantly attenuated. One possible explanation is that PES was moderately correlated to AHI and mean SpO_2_ in our cohort. As illustrated in Table S9 (see online supplementary material for this table), patients with AHI ≥ 30 also had a markedly higher burden of cardiovascular comorbidities, a factor that strongly influences mortality risk.^25^ In a large cohort of patients with severe OSA, age and comorbid conditions were identified as the crucial mortality predictors.^25^

In contrast, among patients with milder OSA (AHI < 30), median PES appeared to play a greater independent role. Patients in both the third and fourth quartiles had significantly increased mortality risk compared to reference (first quartile) (Table S3; see online supplementary material for this table). This notion aligns with the concept that OSA is a heterogeneous disorder^26^ in which some patients exhibit moderate or mild increase in AHI but could generate large negative intrathoracic pressures leading to increased mortality risk. These patients might be missed by risk stratification focusing only on AHI, underscoring the clinical relevance of measuring respiratory effort.

Our study is the first, to our knowledge, to demonstrate a long-term mortality impact of directly measured respiratory effort during sleep. Despite being the most clinically accurate method to estimate intrathoracic pressure and inspiratory effort,^21^ PES is not commonly used in routine sleep apnea evaluations due to its invasiveness and complexity. Consequently, prior OSA mortality studies have lacked direct measurements of respiratory effort, focusing traditionally on AHI.^26^ However, there is growing recognition that OSA’s harm is not fully captured by AHI alone, and that other physiologic dimensions of sleep apnea can better predict outcomes.^26^ Hypoxic burden has been shown to predict cardiovascular mortality risk more robustly than AHI.^27^^,^^28^ Similarly, metrics such as apnea-hypopnea duration^29^ and the magnitude of heart rate responses to apneas have been linked to cardiovascular morbidity.^30^ Our findings extend this paradigm by highlighting negative intrathoracic pressure swings as another key dimension of OSA severity with prognostic importance. Additionally, we present mean nocturnal SpO_2_ as an important and independent predictor of all-cause mortality.

The fact that higher median PES values were still associated with increased mortality risk in the fully adjusted model (which included AHI and mean SpO_2_) suggests that respiratory effort does carry unique information. Mechanistically, large swings in intrathoracic pressure during obstructed breaths can provoke acute surges in transmural cardiac pressures,^31^ impede venous return, and activate sympathetic reflexes, all of which can contribute to negative outcomes.^32^ These pathways are distinct from the effects of hypoxemia and arousal-mediated sympathetic activation in OSA. Therefore our work complements a more holistic approach to characterizing OSA severity, an approach that includes the negative pressure burden of obstructed breathing in addition to the frequency of apneas and hypoxemia during bedtime.

An interesting finding in our exploratory analysis was the dose-dependent increase in mortality risk with greater depth and duration of PES. Figure 2 visualizes that patients who spent more time with PES exceeding increasing thresholds (10, 20, 30 cmH_2_O, etc.), had progressively higher hazard ratios, with the steepest risk seen beyond about 30 cmH_2_O. This implies that both the intensity of inspiratory effort (how negative the pressure) and its duration (%TiB) are valuable markers. In practical terms, brief, infrequent intrathoracic pressure excursions up to 50 cmH_2_O might be less harmful than spending a large portion of the night generating pressures of that magnitude. Our quartile analysis of %TiB above varying thresholds supports this, as the highest quartile of a certain pressure exposure time carried significantly elevated hazards. These results resonate with clinical observations. Patients with more collapsible airways may generate exceptionally negative intrathoracic pressures during prolonged obstructive events, and our data suggest that this imposes added long-term risk.

In light of these findings, clinical decision-making for OSA may benefit from integrating respiratory effort metrics. Currently, PES monitoring is mostly confined to research, patients in ICUs,^33^^,^^34^ or a diagnosis of upper airway resistance syndrome.^35^ Our results suggest it provides clinically meaningful information for patients with suspected OSA. Whether noninvasive surrogates for PES, such as mandibular movements,^36^ pulse transit time,^37^ thoracoabdominal plethysmography (including paradoxical movements), suprasternal pressure transducers, or diaphragmatic EMG could estimate intrathoracic pressure burden warrants further investigation.^20^

Overall, our study provides evidence that negative esophageal pressure is not just a mechanistic phenomenon, but also a clinically relevant and a prognostic factor. These findings should prompt further research into practical methods to measure and mitigate intrathoracic pressure swings in OSA patients. A more comprehensive OSA severity assessment, including frequency of obstruction (AHI), oxygen desaturation, arousal burden, and respiratory effort, would yield better risk stratification for patients with OSA beyond what can be achieved by focusing on apnea/hypopnea counts alone.

## Limitations

Many of our patients, based on polygraphy results (mainly their AHI values), were treated for their sleep apnea by positive airway pressure. Unfortunately, data regarding PAP treatment and adherence to treatment were not available for this study. Treatment with PAP reduces AHI in OSA patients, reverses hypoxemia, and alleviates negative intrathoracic pressure. However, despite these favorable hemodynamic and respiratory effects,^24^ randomized controlled trials on PAP treatment in patients with OSA^6^ have shown neither consistent nor promising results in reducing mortality among those patients.

Nocturnal hypoxic burden^28^ was not available in our sleep examinations. Neither was subjective sleepiness. We did not have access to data about daytime hypoxemia or diagnoses that lead to daytime hypoxemia except for COPD diagnosis.

The use of respiratory polygraphies instead of polysomnography was also a limitation. Failure to measure sleep is associated with underestimation of the AHI and SpO_2_, as time awake between lights off and on will be counted as sleep time. Additionally, our data could not differentiate how much PES was increased due to elevated respiratory drive, heart failure, sleep-related obstruction, or hyperventilation periods of Cheyne-Stokes breathing. Even so, it is less probable that individuals in this study would have generated higher pressure values during wakefulness. It is more likely that this happened during inspiration against occluded upper airways, which is an indirect sign of muscle relaxation during sleep and upper airway collapse. Post-baseline characterization for polygraphy measurements were unfortunately not possible to extract for our cohort.

Because catheter placement was based on a standardized marker-guided protocol and was not formally validated using physiological positioning tests, PES values may not be directly comparable with values obtained using other catheter systems. Another limitation is that pressure swings may partly reflect body fat distribution in addition to OSA-related respiratory effort. Although we adjusted for BMI, we did not have measures of central adiposity such as waist circumference, abdominal girth, or waist-to-hip ratio. Residual confounding by visceral obesity therefore cannot be excluded.

Comorbidity data at baseline was extracted using ICD-code search in the patients’ medical records, which can be associated with underreporting and underestimating the actual prevalence of diseases.^38^ Prevalence of heart failure^39^ and atrial fibrillation^40^ at baseline was quite consistent with the literature when a match with ICD codes was used, whereas diagnoses like COPD,^41^ chronic kidney disease,^42^ diabetes, and hypertension had lower rates than in previous epidemiological studies. Lower mean age at baseline and significantly lower smoking rates in the Norwegian population in the last 2 decades could explain the lower COPD-prevalence in our cohort.

Another limitation of our study is that mortality was assessed as all-cause death, without specific classification of cardiovascular mortality. This limits our ability to draw conclusions about the exact pathways linking respiratory effort to death.

Multicollinearity was assessed in the fully adjusted linear model, including median PES categories, mean SpO_2_ categories, AHI categories, age, BMI, and comorbidities, using variance inflation factor (VIF). Multicollinearity did not distort the estimates, as VIF was less than 3 for all variables included in the regression model.

## Conclusion

Respiratory effort, estimated by esophageal pressure measurement during sleep, independently predicts all-cause mortality, providing prognostic information beyond traditional markers like AHI and hypoxemia. Additionally, both the intensity of esophageal pressure and the duration of exposure seem to have prognostic meaningful effects on mortality risk. These findings highlight respiratory effort as a key factor in OSA pathophysiology and support its role in the comprehensive clinical assessment of the condition.

## Supplementary Material

aamag344_Supplementary_Data
