# Supplementary material for: Respiratory effort during sleep predicts mortality in patients with suspected obstructive sleep apnea
Source: Am J Respir Crit Care Med. 2026 Jun 29;212(11):2803–11. doi: 10.1093/ajrccm/aamag344 (PMC13721115; doi:10.1093/ajrccm/aamag344)
Supplement: Supplementary file 1 [file aamag344_supplementary_data.zip › Supplementary_material,_AJRCC.docx]

**Respiratory effort during sleep predicts mortality in patients with suspected obstructive sleep apnea**

Hamza Nahoui MD^1,4^. Henrik Schirmer MD PhD^1,4^. Gunnar Einvik MD PhD^2,4^. Tarjei Øvrebotten MD^1,4^. Kristian Berge MD PhD^1,4^. Peder L. Myhre MD PhD^1,4,5^. Haldor Husby^6^. Harald Hrubos-Strøm MD PhD^3,4^.

**Supplementary material**

**Tables:	9	Figures: 8**

**Esophageal Pressure Monitoring**

Figure S1: The catheter

A solid-state Millar-type catheter (non-balloon) was used to monitor intrathoracic pressure during sleep. The catheter is 90 cm long with an outer diameter of 2.0 ± 0.2 mm and a soft tip to facilitate transnasal insertion. It contains two solid-state pressure sensors: one located in the pharynx and one in the esophagus. The distal pressure sensor is positioned 50 ± 1 mm from the tip; the proximal sensor and additional thermistors for temperature monitoring are located further proximally, with sensor positions detailed in Figures. The catheter samples pressure at 4 Hz with a sensitivity <1 cmH₂O and accuracy ±2 cmH₂O.

Figure S2: Catheter detail. From left: marker ring, pressure sensor, temperature sensor.

The sensors are based on a silicon integrated circuit chip with a micromachined diaphragm only a few micrometers thick. Four resistors are diffused into the front of the chip to form a Wheatstone bridge. The rear of the diaphragm is exposed to the internal pressure via the catheter lumen, while the front circuit side is open to ambient pressure. The resulting pressure differential induces minimal deflection of the diaphragm, and due to the piezoresistive effect, this mechanical stress is translated into an electrical signal through a change in resistance. Two diagonally opposite resistors increase in resistance, while the other two decrease, causing detuning of the bridge and generating a precise signal. The elastic properties of the silicon diaphragm ensure exact return to resting position, enabling high-fidelity pressure tracking even at high signal frequencies (>100 kHz). This sensitivity also allows the sensor to function as a microphone.

Figure S3: Locations of sensors and markers

Table S1: ICD-codes used to identify comorbidities present in patients at baseline.

| **Comorbidities** | **ICD-10 Codes** |
| --- | --- |
| Heart failure and related cardiomyopathies | I50.x, I42.x |
| Hypertension | I10, I11, I12, I13, I15 |
| Ischemic heart disease | I20.x, I21.x, I22.x, I23.x, I24.x, I25.x |
| Chronic obstructive pulmonary disease | J41.x, J42.x, J43.x, J44.x |
| Stroke and cerebrovascular diseases | G45.x, G46.x, H34.x, I60.x, I61.x, I62.x, I63.x, I64.x, I65.x, I66.x, I67.x |
| Diabetes | E10, E11, E12, E13, E14 |
| Atrial fibrillation | I48.x |
| Chronic kidney disease | N17.x, N18.x, N19.x |
| Anemia | D50.x, D51.x, D52.x, D53.x, D55.x, D56.x, D57.x, D58.x, D59.x, D60.x, D61.x, D62.x, D63.x, D6.x |
| Dementia | F00.x, F01.x, F02.x, F03.x, F051.x, G30.x, G31.1, G31.8 |
| Myocardial infarction | I21.x, I22.x |
| Cancer | C00.x, C11.x, C22.x, C38.x, C50.x, C62.x, C73.x, C84.x, C01.x, C12.x, C23.x, C39.x, C51.x, C63.x, C74.x, C85.x, C02.x, C13.x, C24.x, C40.x, C52.x, C64.x, C75.x, C86.x, C03.x, C14.x, C25.x, C41.x, C53.x, C65.x, C76.x, C88.x, C04.x, C15.x, C26.x, C43.x, C54.x, C66.x, C77.x, C90.x, C05.x, C16.x, C30.x, C44.x, C55.x, C67.x, C78.x, C91.x, C06.x, C17.x, C31.x, C45.x, C56.x, C68.x, C79.x, C92.x, C07.x, C18.x, C32.x, C46.x, C57.x, C69.x, C80.x, C93.x, C08.x, C19.x, C33.x, C47.x, C58.x, C70.x, C81.x, C94.x, C09.x, C20.x, C34.x, C48.x, C60.x, C71.x, C82.x, C95.x, C10.x, C21.x, C37.x, C49.x, C61.x, C72.x, C83.x, C96.x, C97.x |

_Abbreviations: ICD: International Classification of Diseases._

Table S2: Sleep metrics and clinical characteristics between patients who survived and those who died during follow-up.

| Variable | Survivors | Non-survivors | p-value |
| --- | --- | --- | --- |
| Age (years) | 47 (39–56) | 61 (53–69) | <0.001 |
| BMI (kg/m²) | 28.5 (25.7–32.0) | 28.7 (25.8–32.5) | <0.001 |
| AHI (events/hour) | 9.5 (4.3–20.3) | 16.8 (7.7–31.5) | <0.001 |
| Mean SpO₂ (%) | 94.4 (93.2–95.4) | 93.3 (91.9–94.5) | <0.001 |
| Median PES (cmH₂O) | 12.7 (8.6–18.2) | 14.3 (9.9–19.5) | <0.001 |
| Male sex | 10,706 (73.4%) | 1,150 (76.8%) | 0.004 |
| Heart failure | 310 (2.1%) | 195 (13.0%) | <0.001 |
| Atrial fibrillation | 141 (1.0%) | 71 (4.7%) | <0.001 |
| Myocardial infarction | 167 (1.1%) | 51 (3.4%) | <0.001 |
| Ischemic heart disease | 402 (2.8%) | 153 (10.2%) | <0.001 |
| Stroke | 147 (1.0%) | 73 (4.9%) | <0.001 |
| COPD | 92 (0.6%) | 55 (3.7%) | <0.001 |
| Chronic kidney disease | 53 (0.4%) | 39 (2.6%) | <0.001 |
| Hypertension | 677 (4.6%) | 230 (15.4%) | <0.001 |
| Diabetes | 303 (2.1%) | 107 (7.1%) | <0.001 |
| Anemia | 68 (0.5%) | 22 (1.5%) | <0.001 |
| Cancer | 158 (1.1%) | 67 (4.5%) | <0.001 |
| Dementia | 0 (0.0%) | 6 (0.4%) | <0.001 |

Table S3: Association between median negative esophageal pressure (PES) quartiles and mortality stratified by severe OSA, using Cox regression (adjusted for age, sex, BMI, comorbidities and mean SpO_2_ categories).

| Variable | AHI < 30 HR (95% CI), p-value | AHI > 30 HR (95% CI), p-value |
| --- | --- | --- |
| Q2 vs Q1 | 1.17 (0.98–1.40), p=0.089 | 0.88 (0.56–1.38), p=0.571 |
| Q3 vs Q1 | 1.32 (1.10–1.59), p=0.003 | 0.74 (0.49–1.10), p=0.136 |
| Q4 vs Q1 | 1.35 (1.09–1.68), p=0.006 | 0.70 (0.46–1.02), p=0.067 |

**Subgroup analysis stratified by age:**

In age-stratified analyses, the association between median PES and mortality was more pronounced in patients >50 years, with a significant association in the 3^rd^ quartile (HR: 1.22; 95% CI: 1.02–1.47) compared to 1^st^ quartile, while no significant association was observed in patients <50 years (HR: 1.22; 95% CI: 0.81–1.86) for patients in the same quartile, Table S4.

**Stratification by sex:**

Given the increasing proportion of women across quartiles of median PES, we explored whether the association between median PES and mortality differed by sex. We fitted a multivariable Cox model including an interaction term between PES quartiles and sex, adjusting for age, sex, comorbidities, AHI and mean SpO_2_ (Model 3). There was no evidence of effect modification by sex. The interaction terms between PES quartiles and male sex were all close to 1, and the joint test for interaction was not significant (P-interaction = 0.997). Thus, although women were increasingly represented in higher PES quartiles, the association between PES and mortality appeared similar in women and men.

**Censoring at 10 years of follow-up:**

Sensitivity analyses censoring follow-up at 10 years yielded similar hazard ratio estimates as our main analysis and did not alter the association between median PES and mortality. In both Model 1 and Model 2, patients in 3^rd^ and 4^th^ PES quartiles had significantly increased risk of mortality compared with patients in the 1^st^ quartile, whereas in Model 3, adjusted additionally for AHI and mean SpO₂, only patients in 3^rd^ quartile remained significant association, Table S5.

Table S4: Association between median negative esophageal pressure (PES) quartiles and mortality stratified by age > 50 and ≤ 50 years, using Cox regression (Model 3 adjusted for age, sex, BMI, comorbidities, mean SpO_2_ categories, and AHI categories).

| Variable | HR (95% CI), p-value  >50 years | HR (95% CI), p-value  ≤50 years |
| --- | --- | --- |
| Q2 vs Q1 | 1.17 (0.97–1.41), p=0.085 | 0.98 (0.66–1.45), p=0.927 |
| Q3 vs Q1 | 1.22 (1.01–1.47), p=0.033 | 1.27 (0.86–1.88), p=0.222 |
| Q4 vs Q1 | 1.15 (0.93–1.42), p=0.177 | 1.35 (0.88–2.08), p=0.168 |

Table S5: Association between quartiles of median PES and all‑cause mortality across three adjustment models, censored at 10 years of follow up.

| Variable | Model 1(Adjusted for age, sex, BMI) | Model 2(+ comorbidities) | Model 3(+ AHI & SpO₂) |
| --- | --- | --- | --- |
| Q2 vs Q1 | 1.14 (0.96–1.36), p = 0.145 | 1.17 (0.98–1.39), p = 0.091 | 1.15 (0.96–1.38), p = 0.130 |
| Q3 vs Q1 | 1.33 (1.12–1.57), p = 0.001 | 1.30 (1.10–1.53), p = 0.002 | 1.21 (1.02–1.43), p = 0.030 |
| Q4 vs Q1 | 1.38 (1.15–1.64), p < 0.001 | 1.37 (1.14–1.64), p < 0.001 | 1.17 (0.95–1.42), p = 0.136 |

Table S6: Association between AHI categories and all‑cause mortality across three adjustment models. The reference category is mild OSA (AHI < 5). The increased mortality risk observed for severe AHI in Models 1 and 2 was attenuated and no longer statistically significant after full adjustment in Model 3.

|  | Model 1 Adjusted for age, sex, BMI) | Model 2 (+ comorbidities) | Model 3 (+ median PES & SpO₂) |
| --- | --- | --- | --- |
| Mild OSA | 0.90 (0.76–1.06), p=0.216 | 0.90 (0.77–1.07), p=0.255 | 0.88 (0.74–1.04), p=0.146 |
| Moderate OSA | 1.06 (0.90–1.26), p=0.453 | 1.06 (0.89–1.25), p=0.504 | 0.99 (0.83–1.18), p=0.936 |
| Severe OSA | 1.26 (1.05–1.50), p=0.010 | 1.22 (1.02–1.46), p=0.024 | 1.04 (0.85–1.263), p=0.690 |

Table S7: Association between Mean SpO_2_ categories and all‑cause mortality across three adjustment models. The reference category is mean SpO_2_ > 95%. The increased mortality risk observed for mean SpO_2_ in all adjustment models.

|  | Model 1 Adjusted for age, sex, BMI) | Model 2 (+ comorbidities) | Model 3 (+ AHI & median PES) |
| --- | --- | --- | --- |
| Mean spO_2_ <92% | 2.06 (1.72–2.47), p < 0.001 | 1.96 (1.63–2.35), p < 0.001 | 1.82 (1.50–2.21), p < 0.001 |
| Mean SpO_2_ 92-95% | 1.14 (0.98–1.32), p = 0.095 | 1.14 (0.98–1.32), p = 0.095 | 1.10 (0.94–1.28), p = 0.227 |

Table S8: Association between percentage of time in bed (categorized in quartiles) spent above different PES thresholds and mortality risk. Estimated are based on series of Cox regression models adjusted for age, sex, BMI and comorbidities, Model 2.

| %TiB PES | Q2 vs Q1 | Q3 vs Q1 | Q4 vs Q1 |
| --- | --- | --- | --- |
| >10 cmH₂O | 1.17 (0.99–1.37), p = 0.066 | 1.21 (1.03–1.43), p = 0.019 | 1.38 (1.17–1.64), p < 0.001 |
| >20 cmH₂O | 1.34 (1.13–1.58), p = 0.001 | 1.39 (1.18–1.64), p < 0.001 | 1.49 (1.25–1.78), p < 0.001 |
| >30 cmH₂O | 1.19 (1.01–1.42), p = 0.040 | 1.46 (1.24–1.72), p < 0.001 | 1.54 (1.30–1.83), p < 0.001 |
| >40 cmH₂O | 1.03 (0.87–1.21), p = 0.751 | 1.34 (1.14–1.56), p < 0.001 | 1.54 (1.31–1.81), p < 0.001 |
| >50 cmH₂O | 1.17 (0.99–1.38), p = 0.058 | 1.51 (1.30–1.76), p < 0.001 | 1.66 (1.43–1.92), p < 0.001 |
| >60 cmH₂O | 1.11 (0.91–1.35), p = 0.299 | 1.48 (1.29–1.69), p < 0.001 | 1.59 (1.39–1.83), p < 0.001 |

Table S9: Clinical characteristics in patients with or without severe obstructive sleep apnea.

| Variable | No Severe OSA (n=13,464) | Severe OSA (n=2,619) | Total (n=16,083) |
| --- | --- | --- | --- |
| Age (years, median [IQR]) | 47 [39–56] | 53 [44–61] | 48 [40–57] |
| Female sex | 3,738 (27.8%) | 489 (18.7%) | 4,227 (26.3%) |
| Male sex | 9,726 (72.2%) | 2,130 (81.3%) | 11,856 (73.7%) |
| BMI (kg/m², median [IQR]) | 28 [25.4–31.2] | 31.8 [28.4–35.9] | 29.4 [26.1–33.6] |
| Mean SpO₂ (% median [IQR]) | 94.5 [93.5–95.5] | 92.8 [91.3–94.0] | 94.0 [93.2–95.4] |
| Median PES (cmH₂O, median [IQR]) | 11.7 [8.3–16.2] | 22.1 [16.2–29.6] | 14.6 [9.6–20.4] |
| Heart failure | 349 (2.6%) | 156 (6.0%) | 505 (3.1%) |
| Atrial fibrillation | 150 (1.1%) | 62 (2.4%) | 212 (1.3%) |
| Myocardial infarction | 153 (1.1%) | 65 (2.5%) | 218 (1.4%) |
| Ischemic heart disease | 399 (3.0%) | 156 (6.0%) | 555 (3.5%) |
| Stroke | 161 (1.2%) | 59 (2.3%) | 220 (1.4%) |
| COPD | 117 (0.9%) | 30 (1.1%) | 147 (0.9%) |
| Chronic kidney disease | 58 (0.4%) | 34 (1.3%) | 92 (0.6%) |
| Hypertension | 639 (4.7%) | 268 (10.2%) | 907 (5.6%) |
| Diabetes | 291 (2.2%) | 119 (4.5%) | 410 (2.5%) |
| Anemia | 64 (0.5%) | 26 (1.0%) | 90 (0.6%) |
| Cancer | 168 (1.2%) | 57 (2.2%) | 225 (1.4%) |

Figure S4: Histogram displaying the distribution of median PES across the study population. The variable shows a right skewed (positively skewed) distribution, with most patients clustering at lower PES values and a long tail extending toward higher values. While the majority of individuals experienced relatively modest negative pressures, a smaller subset experienced markedly elevated respiratory effort during sleep.

Figure S5: Scatterplot illustrating the correlation between Apnea Hypopnea Index (AHI) and median negative esophageal pressure (PES). Each point represents an individual participant. A positive, moderately strong correlation is observed, as indicated by the upward trend of the fitted regression line in red. This suggests that higher AHI values are generally associated with more negative (i.e., greater magnitude) esophageal pressures.

Figure S6: Scatterplot illustrating the correlation between mean SpO₂ (%) and median PES (cmH₂O). Each point represents an individual patient. A moderate inverse correlation is observed, with the fitted regression line (red) indicating that lower mean SpO₂ values are associated with more negative (i.e., greater magnitude) median PES. This suggests that increased respiratory effort during sleep is linked to more severe nocturnal hypoxemia

Figure S7: Kaplan-Meier survival curves showing the cumulative survival over time across quartiles of median PES. Patients in higher quartiles exhibited progressively lower survival compared to those in the first quartile. The increased mortality risk is evident starting from the second quartile and becomes more pronounced in the third and fourth quartiles. However, survival curves for the third and fourth quartiles closely overlap, indicating no substantial difference in mortality between these two groups. The divergence in survival begins within the first few years of follow-up and continues to widen over time.

Figure S8: Association between AHI and all-cause mortality using restricted cubic spline analysis. The hazard ratio increases gradually with higher AHI values, although the 95% confidence interval (shaded area) overlaps 1.0 across the AHI spectrum, indicating a non-significant association. The model was adjusted for age, sex, BMI, comorbidities, mean SpO₂, and median negative esophageal pressure, with continuous variables modeled as splines.
